# Predictive value of intravascular ultrasound for the function of intermediate coronary lesions

**DOI:** 10.1186/s12872-023-03489-0

**Published:** 2023-09-14

**Authors:** Yajuan Zhu, Guowei Zhou, Lei Yang, Keng Liu, Yuning Xie, Wen-Yi Yang, Qiuyan Dai

**Affiliations:** 1 Department of Cardiology, Shanghai General Hospital of Nanjing Medical University, No.100, Haining Rd, Hongkou District, Shanghai, 200080 China; 2 Department of Cardiology, Shanghai General Hospital of Shanghai Jiao Tong University School of Medicine, No.100, Haining Rd, Hongkou District, Shanghai, 200080 China; 3 Department of Emergency, Shanghai General Hospital of Shanghai Jiao Tong University School of Medicine, No.100, Haining Rd, Hongkou District, Shanghai, 200080 China; 4 Menghai County People’s Hospital, Xishuangbanna, Yunnan Province China; 5 School of Oral Medicine, Nanjing Medical University, No.1, Shanghai RD, Nanjing City, Jiangsu Province China

**Keywords:** Intravascular ultrasound (IVUS), Contrast-flow quantitative flow ratio (QFR), Logistic regression analysis, ROC curve, Intermediate coronary lesion

## Abstract

**Background:**

Intravascular ultrasound (IVUS) can provide detailed coronary anatomic parameters. The purpose of our study was to evaluate the parameters measured by IVUS for the prediction of intermediate coronary lesions function by referencing quantitative fraction ratio (QFR) ≤ 0.80 (vs. > 0.80).

**Methods:**

Eighty four cases with 92 intermediate coronary lesions in vessels with a diameter ≥ 2.50 mm were enrolled. Paired assessment of IVUS and cQFR was available, and vessels with cQFR ≤ 0.8 were considered the positive reference standard. Logistic regression was used to select model variables by a maximum partial likelihood estimation test and receiver operating characteristic curve (ROC) analysis to evaluate the diagnostic value of different indices.

**Results:**

Plaque burden (PB) and lesion length (LL) of IVUS were independent risk factors for the function of coronary lesions. The predictive probability P was derived from the combined PB and LL model. The area under the curve (AUC) of PB, (minimum lumen area) MLA, and LL and the predicted probability P are 0.789,0.732,0731, and 0.863, respectively (*P* < 0.01). The AUC of the predicted probability P was the biggest among them; the prediction accuracy of cQFR ≤ 0.8 was 84.8%, and the sensitivity of the diagnostic model was 0.826, specificity was 0. 725, and *P* < 0.01.

**Conclusion:**

PB and LL of IVUS were independent risk factors influencing the function of intermediate coronary lesions. The model combining the PB and LL may predict coronary artery function better than any other single parameter.

**Supplementary Information:**

The online version contains supplementary material available at 10.1186/s12872-023-03489-0.

## Introduction

Coronary atherosclerotic heart disease has become a major disease endangering human health. The optimization of drug therapy, development of interventional devices, and advancement of surgical techniques have significantly benefited patients with coronary heart disease. Percutaneous coronary intervention (PCI) has reduced the mortality rate in patients with acute coronary syndrome [1]. However, whether active revascularization therapy is necessary for intermediate lesions has always been a hot topic of debate. Intermediate lesions are defined as those with 50%-70% stenosis on coronary angiography [2]. Whether angina pectoris necessitates revascularization or drug therapy for the stability of intermediate lesions remains controversial; thus, the method for evaluating function is essential.

There is increasing evidence that two-dimensional coronary angiography is limited in determining whether intermediate lesions require only optimal medical therapy or additional stenting. The fractional flow reserve (FFR) serves as the gold standard in terms of assessing coronary flow functionality [3–5]. However, determination of the FFR has disadvantages, such as its invasive nature, associated radiation exposure, side effects from hyperaemic agents, and high costs; as such, it is not commonly used in practical clinical applications [6]. The quantitative flow ratio (QFR) is determined by a novel technique for rapidly evaluating coronary function without additional consumables and medications; additionally, this technique allows retrospective analysis of collected coronary angiography images. Several randomized clinical trials have confirmed its effectiveness and accuracy [7–9]. Compared with the FFR, the QFR has a similar diagnostic accuracy for functional coronary artery disease and is a reliable metric for assessing coronary haemodynamics [10–12]. Determination of the QFR is accepted as a rapid, drug-free method for assessing coronary function without additional supplies.

IVUS can provide information on vascular anatomy, but there is a need for improvement regarding indications of coronary function. This study aimed to accurately predict coronary function according to IVUS parameters.

## Methods

### Study design and populations

**Fig. 1:**
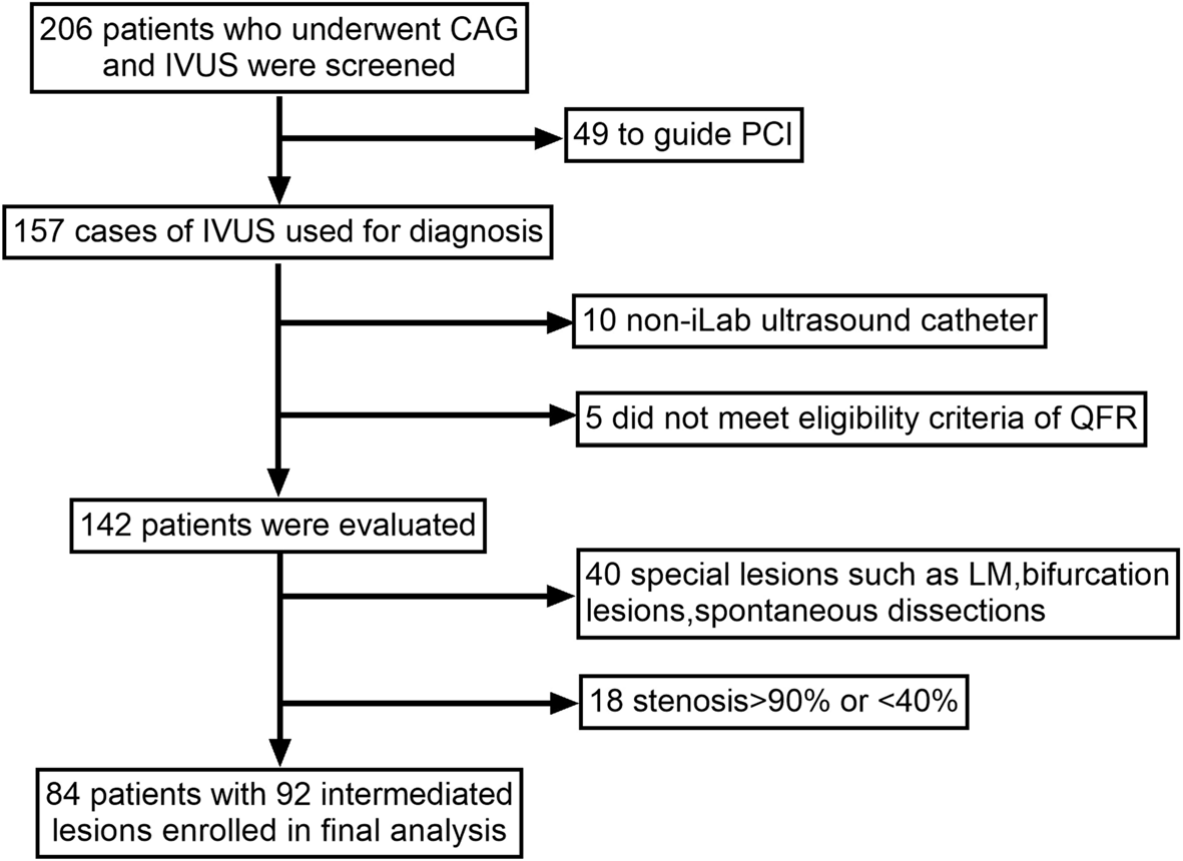
Flowchart of patients enrolled

This was a retrospective analysis of patients referred for coronary angiography from January to December 2020 at Shanghai General Hospital affiliated with Shanghai Jiao Tong University. In all, 206 consecutive patients with stable angina, unstable angina, and asymptomatic myocardial ischaemia were enrolled. According to the prespecified protocol, 84 patients with 92 intermediate coronary lesions in vessels with a diameter ≥ 2.50 mm were included in the final analysis (Fig. 1). All patients signed a preoperative informed consent form. The exclusion criteria were as follows: (1) iodine contrast allergy; (2) history of malignancy or autoimmune disease; (3) pregnancy or lactation; (4) graft vascular disease; (5) chronic occlusive lesions; (6) haemodynamic instability; and (7) NYHA IV heart failure resulting in inability to tolerate the procedure.

All patients undergoing coronary angiography were given preoperative loads of clopidogrel (or ticagrelor) and aspirin, signed an informed consent form before coronary angiography, and were given secondary prevention medications such as statins, β-blockers, angiotensin-converting enzyme inhibitors (ACE-Is), angiotensin receptor blockers (ARBs)/calcium channel blockers, and other coronary drugs depending on their condition.

The right radial artery was preferred for puncture access by the percutaneous coronary intervention physician, and conventional multiposition projection was performed in all enrolled patients. An intermediate lesion was defined by greater than 50% stenosis. The evaluated vessels included the LAD, LCX, RCA, and branch vessels ≥ 2.5 mm in diameter, in addition to the LM.

### IVUS imaging

**Fig. 2:**
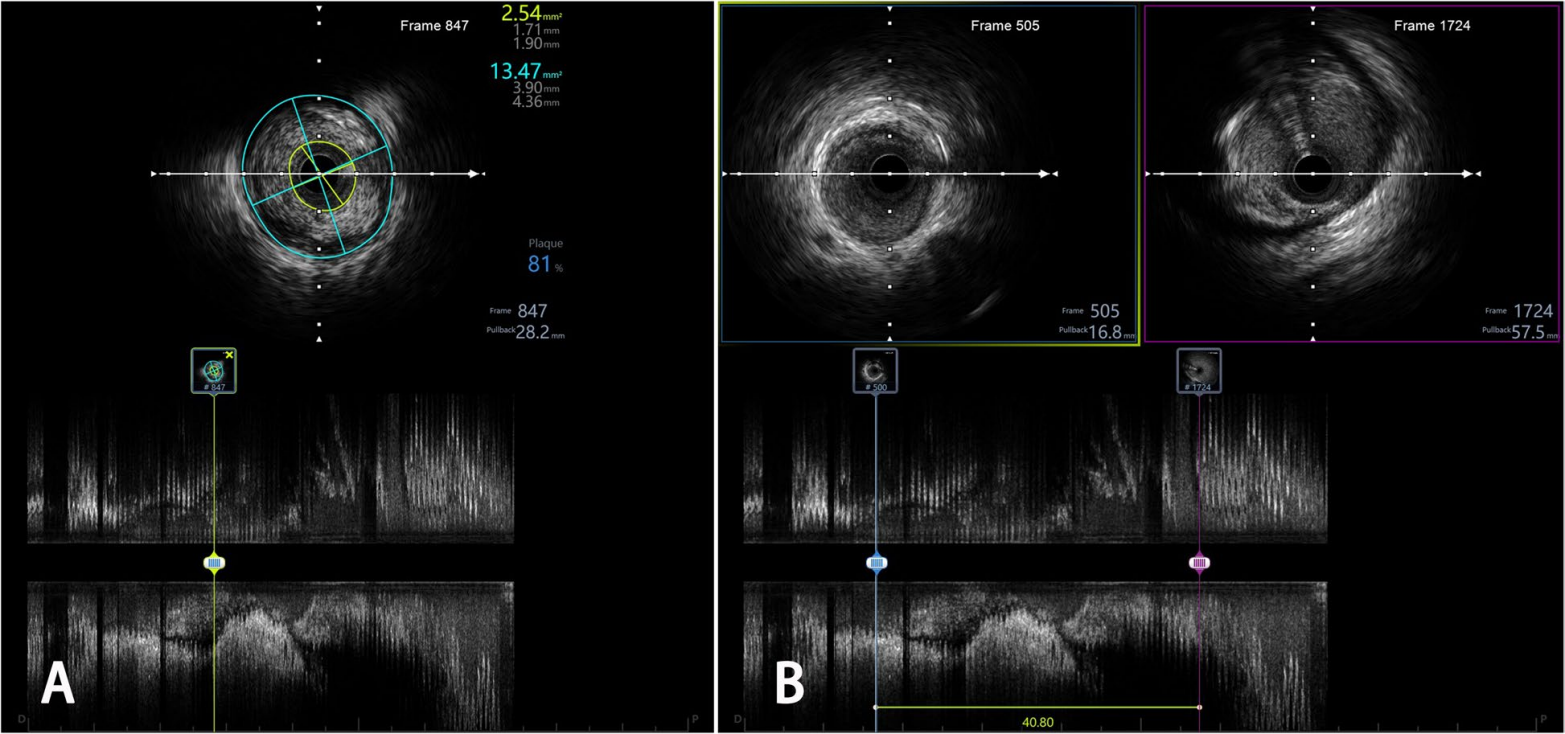
IVUS measured the short and long axis of the vascular lesion. **A** The MLA of the lesion was 2.54mm^2^ on the short-axis image, and the PB was 81%. **B** From the proximal normal segment of the lesion to the distal in a long-axis diagram, the LL of the vessels was 40.8 mm

Intravascular ultrasound (IVUS) studies were performed using a 3.2 F catheter containing a single rotating element transducer of 40 MHz connected to an IVUS system (iLab, Boston Scientific Corp., CA, USA). Before image acquisition, we performed an intracoronary infusion of 100–200 μg of nitroglycerine to avoid vessel spasms. After the ultrasound catheter was advanced 10 mm distal to the target lesion, it was automatically pulled back at 0.5 or 1.0 mm/s to the coronary ostium. All images were recorded for offline analysis. iLab review software was applied to measure the minimum lumen area (MLA), the cross-sectional area of the external elastic membrane, the diameter of the proximal and distal reference lumen, the lumen area, the plaque burden (PB), and the lesion length (LL) of the target coronary vessel. The lesion was defined by the smallest cross-sectional lumen area. The PB was defined as follows: (external elastic membrane cross-sectional area—minimum lumen area)/external elastic membrane cross-sectional area. A long-axis image of the diseased vessel was obtained from the IVUS diagram, and the cursor was moved to the proximal start point and distal end point of the lesion. The vascular LL could be measured by dragging the marker from the proximal start point to the distal end point. The IVUS measurement method is demonstrated in Fig. 2.

### Offline QFR assessment

**Fig. 3:**
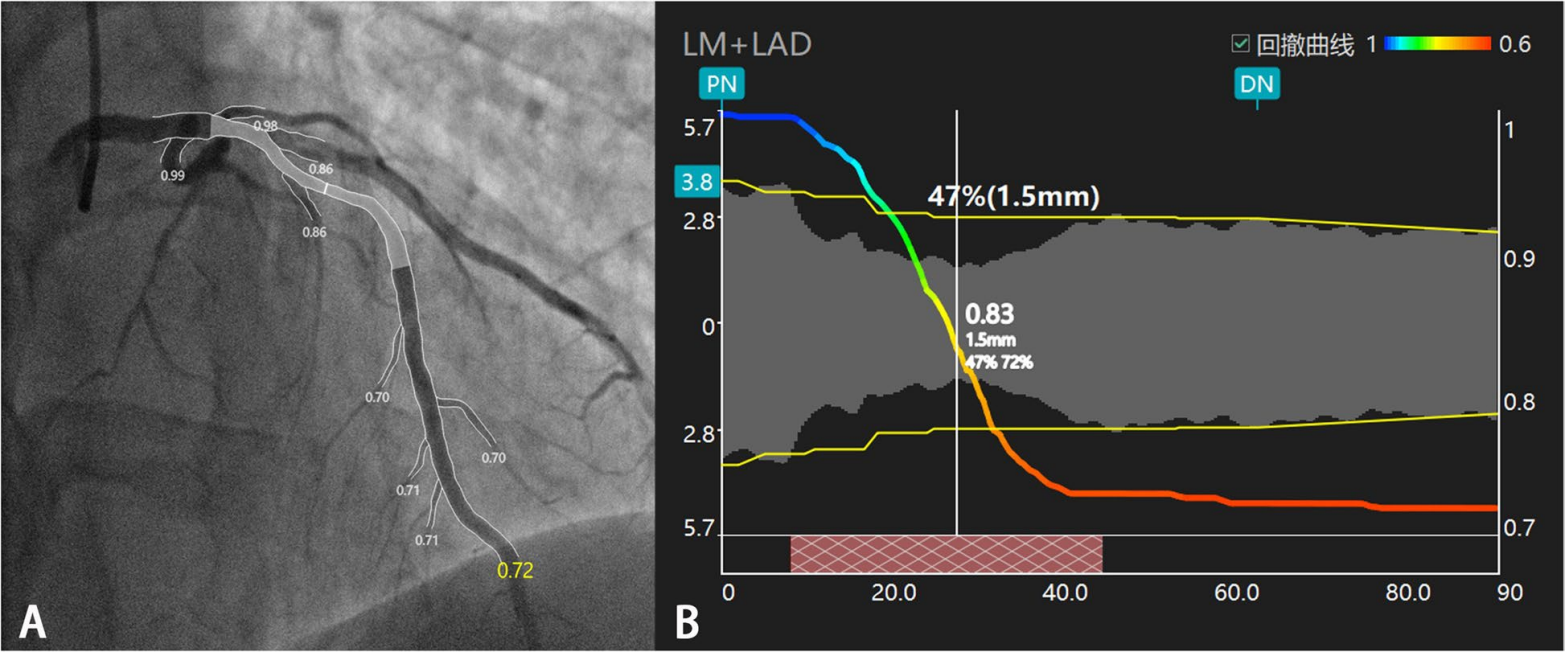
**A** The contrast QFR(QFR) of the LAD vessels was 0.72, the lesion was located in the middle segment of LAD, and the QFR values of each branch were also shown. **B** Testing QFR on the long axis of vessels with 2D, diameter stenosis (DS) was 47%, and area stenosis (AS) was 72%

The QFR was analysed by two qualified physicians using QFR system software (AngioPlus, Pulse Medical Imaging Technology, Shanghai Co., Ltd., Shanghai, China). We imported the contrast images of enrolled patients and selected the frame that most clearly showed the affected vessel (especially the lesion). We first identified the vessel, e.g., the LAD, LCX, or RCA. The keyframe, which presented the least foreshortening of the stenotic area and minimum overlap of the main vessel and side branches, was used for analysis. The investigator identified two anatomic landmarks (e.g., bifurcations) as reference points and indicated the most proximal and distal sites of the vessel. Vessel contours were automatically detected and manually corrected if needed. Automatic detection is better than manual correction; thus, artificial correction was avoided as much as possible. When the diseased segment was given priority, we ensured that the reference lumen of the normal vascular segment was consistent with the actual lumen. The 2D QFR calculation was completed for the main and side branches, and the reports were stored. Figure 3 illustrates measurement of the QFR of the LAD.

### Statistical analysis

Statistical software SPSS version 25 was applied for data input and analysis. The measurement data were tested for normality using the Shapiro‒Wilk test. Continuous variables with a normal distribution are presented as the mean ± standard deviation $\left(\overline{\mathrm{x}}\pm \mathrm{s }\right)$. The t test was used for comparisons between two groups, one-way ANOVA was used for comparisons among multiple groups, and the LSD t test was used for two-by-two comparisons. Categorical variables are presented as counts and percentages. Variables that did not conform to a normal distribution are expressed as the median (M) and interquartile range (Q1-Q3). For nonparametric data, the Mann‒Whitney U test was used for comparisons between two groups, the Kruskal‒Wallis H test was used for comparisons among multiple groups, and the Nemenyi test was used for two-by-two comparisons. The Pearson correlation test was used for the univariate correlation analysis of continuous variables, and the Spearman correlation test was used for the analysis of nonnormally distributed or ranked data. Data were divided into groups according to QFR ≤ 0.80 and QFR > 0.80. Univariate logistic regression was used to select clinical risk factors for clinical model establishment. Clinical features with *P* < 0.05 were included in a multivariate binary logistic regression analysis to build a clinical model. The maximum likelihood ratio probability test was used to select the independent variables, and differences were considered statistically significant at *P* < 0.05 to screen out factors affecting the QFR. The main influencing factors were filtered out, and the predicted probability P was derived. Receiver operating characteristic (ROC) curves were generated to assess the area under the curve (AUC) of the MLA, PB, LL, and probability P for predicting QFR ≤ 0.8. A *P* value < 0.05 was considered to indicate statistical significance.

## Results

### Patient characteristics

The patient's baseline characteristics and lesion characteristics are shown in Table 1.

**Table 1:** Patients' baseline characteristics and lesion characteristics (*n* = 84)

General	
Age (years)	67.6 ± 9.9
Male, cases (%)	59 (70%)
BMI, kg/m^2^	24.2 ± 3.6
SBP, mmHg	135.5 ± 20.5
DBP, mmHg	78.1 ± 11.5
Medical history	
Hypertension, cases (%)	51 (61%)
Diabetes mellitus, cases (%)	25 (30%)
Smoking history, cases (%)	17 (20%)
History of alcohol consumption, cases (%)	7 (8%)
Clinical diagnosis	
Stable angina pectoris, cases (%)	37 (44%)
Unstable angina pectoris, cases (%)	17 (20%)
Asymptomatic myocardial ischemia, cases (%)	30 (36%)
Concomitant medication	
Aspirin, cases (%)	84 (100%)
Ticagrelor or clopidogrel, cases (%)	84 (100%)
Statin, cases (%)	76 (91%)
Beta-blocker, cases (%)	54 (64%)
ACE-I/ARB, cases (%)	56 (67%)
CCB, cases (%)	37 (44%)
Nitrates, cases (%)	18 (21%)
Trimetazidine, cases (%)	9 (11%)
Other medicine, cases (%)	5 (6%)

*BMI* Body mass index, *SBP* Systolic blood pressure, *DBP* Diastolic blood pressure, *ACE-I* Angiotensin-converting enzyme inhibitor, *ARB* Angiotensin receptor blocker, *CCB* Calcium channel blockers

### Lesion characteristics

Ninety-two lesions in 84 patients, including 71 in the left anterior descending (LAD) artery, 10 in the left circumflex (LCX) artery, and 11 in the right coronary artery (RCA), were finally analysed. There were 23 vessels with a contrast-flow quantitative flow ratio (QFR) ≤ 0.8 and 69 vessels with a QFR > 0.8. The mean diameter of stenosis (DS) at the baseline target vessel lesion was 40.11 ± 10.41%, and the mean area of stenosis (AS) at the vessel lesion was 62.03 ± 12.31%, as measured by QCA. The median MLA measured by IVUS at the corresponding target vascular lesion was 3.80 (3.03–4.91) mm2 (nonnormal distribution), and this value was lower in those with a history of hypertension (*n* = 54) than in those without hypertension (*n* = 38) [3.70 (2.97–4.40) vs. 4.01 (3.48–5.69), *P* = 0.035]. The median MLA was also lower in those with a smoking history (*n* = 19) than in those without a smoking history (*n* = 73) [3.36 (2.54–4) vs. 3.81 (3.32–5.45), *P* = 0.023]. Nevertheless, there were no significant differences in these observations by age, sex, history of diabetes or alcohol consumption, BMI, or target blood vessels. (Table S1).

The plaque burden (PB) was 70% (62–74%) in those with a history of hypertension (*n* = 54), higher than that in those without hypertension (*n* = 38) [70% (63.8–76%) vs. 66% (50–73.3%), *P* = 0.038]. The PB was higher in those with a history of diabetes (*n* = 27) than in those without diabetes (*n* = 65) [74% (65–77%) vs. 68% (56–72%), *P* = 0.025]. There was no significant difference in the PB by age, sex, history of smoking or alcohol consumption, BMI, target vessels, or other characteristics. (Table S2).

The LL on IVUS was 18.5 (13.6–27.9) mm. There was no significant difference in the LL by age, sex, history of hypertension, diabetes, smoking, or alcohol consumption, BMI, target vessels, or other characteristics.

### QFR in different states

The median QFR was 0.88 (0.80–0.93). The QFR was lower in those with a history of hypertension (*n* = 54) than in those without hypertension (*n* = 38) [0.86 (0.76–0.91) vs. 0.91 (0.84–0.95), *P* = 0.023]. On the other hand, there was no significant difference in the QFR by age, sex, history of hypertension, diabetes, smoking, or alcohol consumption, BMI, or target vessels, as shown in Table 2.

**Table 2:** Comparison of cQFR in different states

Variables	cQFR	Z or H value	*P*-Value
Age, yrs		-0.257	0.797
< 60 (*n* = 17)	0.88(0.73–0.94)		
≥ 60 (*n* = 75)	0.87(0.81–0.93)		
Gender		-1.064	0.287
Male (*n* = 65)	0.89(0.82–0.94)		
Female (*n* = 27)	0.85(0.74–0.92)		
History of HTN		-2.282	0.023
Yes (*n* = 54)	0.86(0.76–0.91)		
None (*n* = 38)	0.91(0.84–0.95)		
History of DM		-1.42	0.156
Yes (*n* = 27)	0.85(0.74–0.93)		
None (*n* = 65)	0.89(0.81–0.94)		
History of smoking		-1.902	0.057
Yes (*n* = 19)	0.85(0.72–0.88)		
None (*n* = 73)	0.89(0.81–0.94)		
History of alcohol		-0.361	0.718
Yes (*n* = 7)	0.88(0.72–0.93)		
None (*n* = 85)	0.88(0.805–0.94)		
BMI		-0.214	0.830
< 24 (*n* = 43)	0.88(0.8–0.94)		
≥ 24 (*n* = 47)	0.88(0.77–0.93)		
Vascular		1.582	0.453
LAD (*n* = 71)	0.86(0.76–0.93)		
LCX (*n* = 10)	0.92(0.86–0.96)		
RCA (*n* = 11)	0.92(0.87–0.95)		

*cQFR* Contrast quantitative flow ratio, *HTN* Hypertension, *DM* Diabetes mellitus, *BMI* Body mass index, *LAD* Left anterior descending coronary artery, *LCX* Left circumflex coronary artery, *RCA* Right coronary artery

### Association between QFR and IVUS indices

**Fig. 4:**
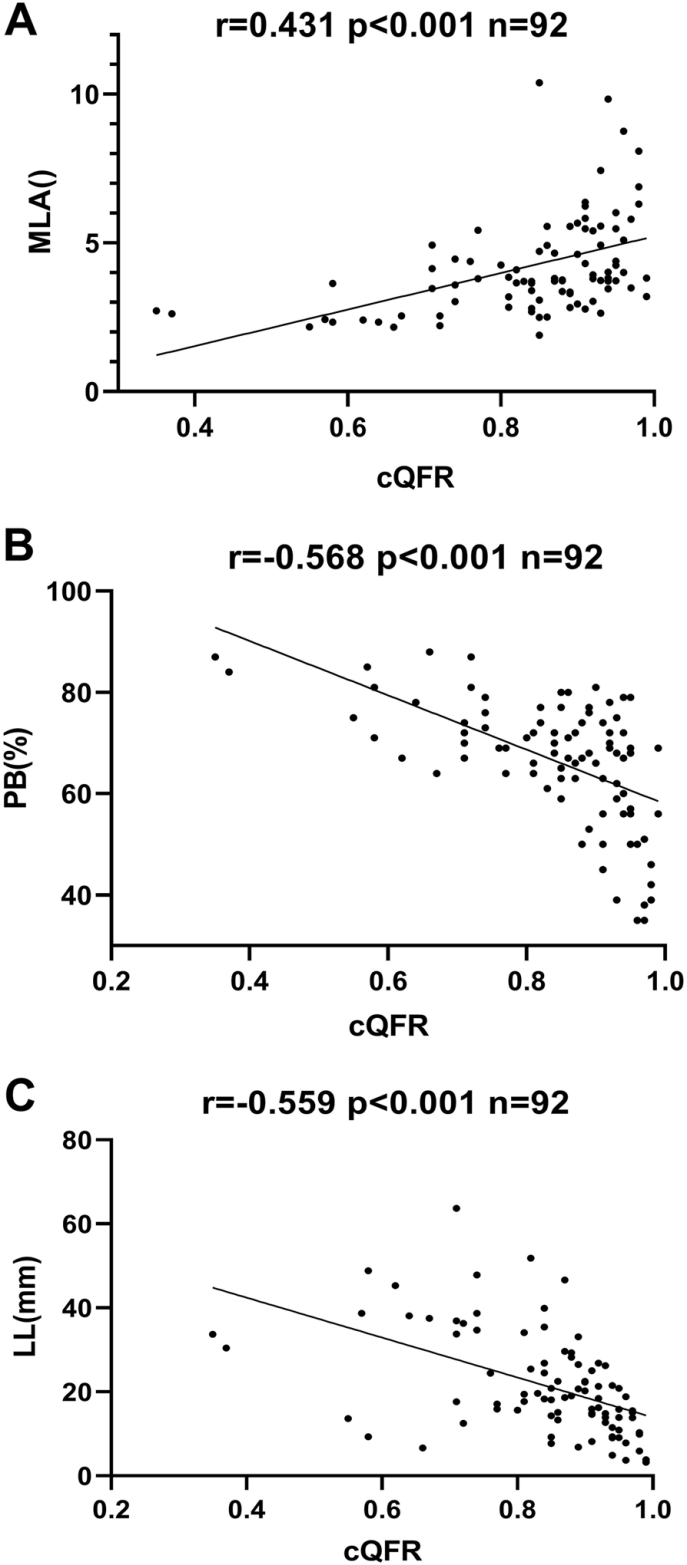
**A** Correlation analysis of QFR and MLA; **B** Correlation analysis of QFR and PB; **C** Correlation analysis of QFR and LL

The QFR positively correlated with the MLA at the corresponding target vessel lesion site (*r* = 0.431, *P* < 0.001) (Fig. 4A), negatively correlated with the PB at the lesion site (*r* = -0.568, *P* < 0.001) (Fig. 4B), and negatively correlated with the LL at the lesion site (*r* = -0.559, *P* < 0.001) (Fig. 4C). According to the data analysis, it was clear that although the QFR was negatively correlated with the PB, the correlation between the QFR and PB was the most potent among the correlations with the PB, MLA, and LL.

The patients were divided into two groups based on a cut-off value of 0.8, i.e., QFR ≤ 0.80 and QFR > 0.80. Lesions with QFR ≤ 0.8 showed lower MLA and higher PB, LL compared with QFR > 0.8 (MLA: 3.02(2.4–4.14) vs. 3.81(3.375–5.51), *P* = 0.001; PB: 74% (69–81)% vs. 66% (56–72)%, *P* < 0.001). LL was more severe in the group of QFR ≤ 0.8 (LL: 33.8(15.9–38.7) vs. 17.7(12.1–22.5), *P* = 0.001) (Table 3). Binary logistic regression analysis was performed to determine the relationship between the MLA, PB, and LL measured on IVUS between the two groups. The results showed that the PB had a significant effect on QFR ≤ 0.8 (*P* = 0.007, crude OR = 1.163). The LL significantly affected QFR ≤ 0.8 (*P* = 0.002, crude OR = 1.079). The regression coefficient of the MLA for QFR ≤ 0.8 was negative and not statistically significant (*P* = 0.871) (Table 4).

**Table 3:** Lesion characteristics between the QFR ≤ 0.8 and the QFR > 0.8

	**QFR ≤ 0.8(*N* = 23)**	**QFR > 0.8(*N* = 69)**	**Z**	**P**
MLA (mm2)	3.02 (2.4–4.14)	3.81 (3.375–5.51)	-3.323	0.001
PB (%)	74 (69–81)	66 (56–72)	-4.133	< 0.001
LL (mm)	33.8 (15.9–38.7)	17.7 (12.1–22.5)	-3.309	0.001

**Table 4:** Logistics regression analysis of the effect of MLA, PB, and LL on QFR

	**B**	**SE**	**Wald**	**P**	**OR**	**CI 95%**	
PB	0.151	0.056	7.207	0.007	1.163	1.042	1.299
length	0.076	0.025	9.434	0.002	1.079	1.028	1.133
MLA	-0.060	0.372	0.026	0.871	0.941	0.454	1.950
Constants	-13.457	5.202	6.692	0.010	0.000	-	-

*B* Regression coefficient, *SE* Standard error, *Wald* Chi-squared value, -. No data is available, *OR* Odds ratio, *CI* Confidence interval

To further eliminate the interfering factors and evaluate the ability of IVUS variables to predict coronary function, we analysed the correlations between the MLA, PB, and LL and the presence of significant vascular function. We set QFR ≤ 0.8 and QFR > 0.8 as dependent variables (QFR ≤ 0.8 is 1, QFR > 0.8 is 0), and established a logistic regression model to evaluate the optimal measurement variables on IVUS. Each indicator with statistical significance was taken as an independent variable, and the maximum likelihood estimation test (forwards: LR) was performed. The PB, MLA, and LL were screened and entered into a regression model, and the overall model predicted 84.8% of coronary function. The PB and LL were positively correlated with QFR ≤ 0.8, and the system automatically excluded MLA, which was not statistically significant. The prediction model was based on the screened variables, as follows: Logit(p) = ln[P/(1-P)] = -14.079 + 0.156 × PB + 0.077 × LL. According to the Wald value, the factor with the most significant influence on the QFR was the PB, followed by the LL (*P* < 0.05) (Table 5).

**Table 5:** Variables in the equation

		**B**	**SE**	**Wald**	***P***	**OR**	**95%CI**	
**step 1a**	PB	0.141	0.039	13.338	< 0.001	1.152	1.068	1.242
	Constants	-11.034	2.825	15.261	< 0.001	0	-	-
**step 2b**	PB	0.156	0.046	11.407	0.001	1.169	1.068	1.28
	length	0.077	0.025	9.865	0.002	1.08	1.029	1.133
	Constants	-14.079	3.61	15.21	< 0.001	0	-	-
a Variable entered at step 1: PB								
b Variable entered at step 2: lesion length								

*B* Regression coefficient, *SE* Standard error, *Wald* Chi-squared value, -. *No data* is available, *OR* Odds ratio, *CI* Confidence interval

**Fig. 5:**
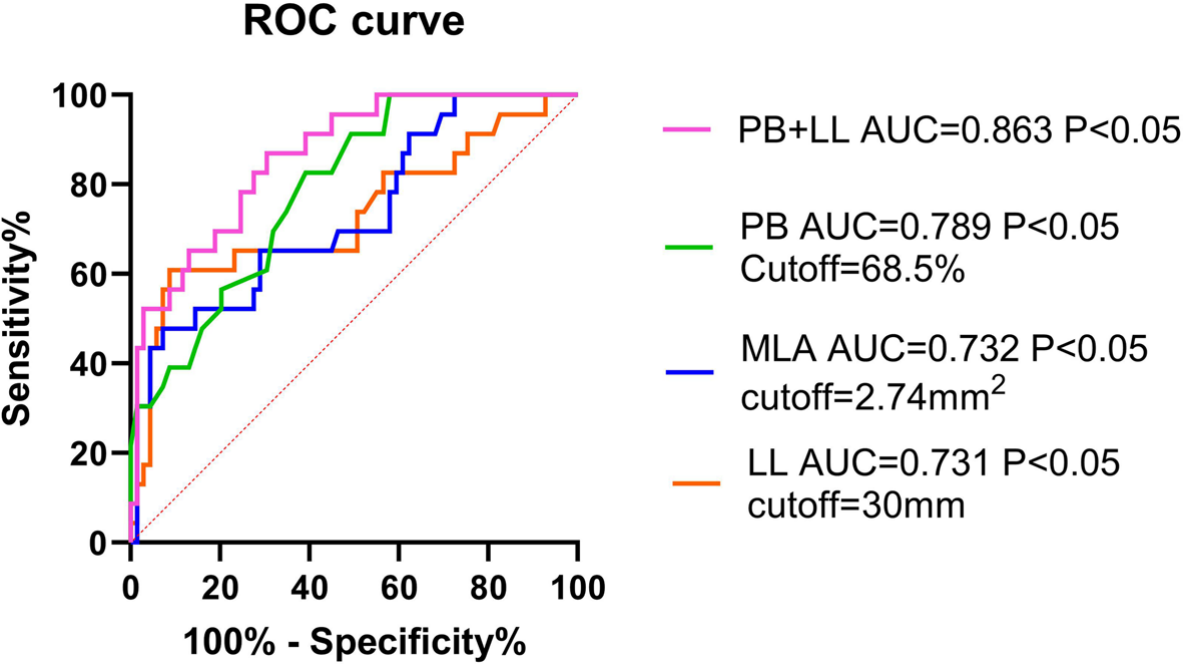
ROC curve for predicted QFR ≤ 0.80

We established the ROC curve and calculated the area under the curve (AUC) to demonstrate the diagnostic efficacy of the PB, MLA, LL, and predicted probability P (the predicted probability P was the variable obtained after the above logistic regression including the PB and LL, PB + LL). The AUCs of the PB, MLA, LL and PB + LL were 0.789, 0.732, 0731, and 0.863, respectively, among which the AUC of PB + LL was the largest. The best PB, MLA, and LL cut-off values for predicting QFR ≤ 0.8 were 68.5%, 2.74 mm2, and 30 mm, respectively, as shown in Fig. 5. The positive predictive values (PPVs) of the PB, MLA, LL, and PB + LL were 0.928, 0.957, 0.986, and 0.957, respectively. The negative predictive values (NPVs) of the PB, MLA, LL, and PB + LL were 0.348, 0.391, 0.217, and 0.522, respectively. This combined diagnostic model had a sensitivity of 0.87, a specificity of 0.696 (*p* < 0.001), an accuracy of 84.8%, and a Jorden index r of 0.566 (Table 6).

**Table 6:** Diagnostic value of different variables

	**AUC**	**cut-off**	**Sen**	**Spe**	**AC**	**PPV**	**NPV**	**Youden**	**SE**	***P***	**95%CI**	
PB	0.789	> 68.5	0.826	0.609	78.30	0.928	0.348	0.435	0.05	< 0.001	0.691	0.886
MLA	0.732	< 2.74	0.478	0.928	79.30	0.957	0.391	0.406	0.062	0.001	0.61	0.854
LL	0.731	> 30	0.609	0.913	81.50	0.986	0.217	0.522	0.069	0.001	0.597	0.866
PB + LL	0.863	0.196	0.87	0.696	84.80	0.957	0.522	0.566	0.041	< 0.001	0.783	0.943

*AUC* The area under the curve, *Sen* Sensitivity, *Spe* Specificity, *AC* Accuracy, *PPV* Positive prediction value, *NPV* Negative prediction value, *SE* Standard error, *CI* Confidence interval

## Discussion

The diagnostic efficacy of single parameters such as PB, MLA, or LL is unsatisfactory. A new joint parameter, PB + LL, is introduced. The combination of PB and LL can improve the value of IVUS parameters in predicting coronary artery function.

Determination of the quantitative flow ratio (QFR) is a new functional method to evaluate whether sites of coronary artery stenosis are of haemodynamic significance. This method is based on 3D angiographic reconstruction and calculation of the FFR via fluid dynamics and thus requires no additional drug injections or pressure wires. The QFR has received much attention in previous clinical trials due to its availability. The FAVOR Pilot and FAVOR II studies are the first studies to demonstrate that the QFR, measured without pressure wires, is superior to data obtained by standard quantitative coronary angiography in evaluating intermediate coronary lesions [7, 13]. The primary endpoint, the per-vessel diagnostic accuracy of the QFR, was 92.7%, which was significantly higher than the protocol-specified target value [8]. In addition, compared with the FFR, the QFR is accurate in diagnosing functional coronary artery disease and is a reliable metric for assessing coronary haemodynamics [10–12]. In a multicentre, randomized, sham-controlled trial in patients with coronary artery disease undergoing PCI, a QFR-guided vessel and lesion selection strategy improved 1-year clinical outcomes compared with standard angiography guidance [9]. Recently, the QFR has been found to be feasible for selecting patients for FFR referral [14]; Paweł confirmed the good diagnostic performance of the QFR and its correlation with the iFR for detecting the functional ischaemia caused by intermediate lesions in coronary arteries [15, 16]. The WIFI-II study showed that functional lesion evaluation by QFR measurement is feasible and shows good agreement and diagnostic accuracy compared with the FFR in patients with intermediate stenosis [11]. The European Association of Percutaneous Cardiovascular Interventions (EAPCI) recently indicated that the QFR is the only angiography-based physiological index that has been prospectively validated and is associated with improved clinical outcomes when used to decide upon coronary revascularisation compared with conventional angiography [17]. Therefore, using the QFR as a reference index for vascular function assessment is a reasonable strategy.

In clinical practice, IVUS is used to accurately determine the nature and degree of coronary lesion stenosis, guide stent implantation, and evaluate stent apposition after stenting. However, the definitions of parameters for detecting functional intermediate lesions remain controversial. A prospective study suggested that IVUS-MLA ≤ 4 mm2 could be considered to indicate functional stenosis, which may require revascularization [18]. The vessel size should always be taken into account when determining the MLA associated with active ischaemia, and ROC analysis identified the best threshold value for FFR < 0.8 as MLA < 3.6 mm2 (AUC = 0.70) in lesions with a reference vessel diameter > 3.5 mm [19]. However, more recently, it has been found that there is considerable heterogeneity in the MLA-based prediction of functional significance in nonprincipal lesions, with actual thresholds ranging from 2.3 to 4.0 mm2. In contrast, both thresholds have limited accuracy [20, 21]. Additionally, in the present study, we found that the correlation between the MLA and QFR was not significant, which may be related to the lack of segmental vascular differentiation. The QFR showed a moderate correlation with the MLA (MLA: *r* = 0.431, *P* < 0.001). In previous studies on IVUS and the FFR, the PB predicted FFR thresholds fluctuating from 65 to 75% [22–24], which is generally consistent with the results of the present study.

The use of one IVUS parameter alone yields only limited diagnostic efficacy. We found that the diagnostic efficacy of a single parameter, such as the PB, MLA, or LL, was unsatisfactory. The AUC for predicting coronary function with PB > 68.5% was 0.789, with an accuracy of 78.3% and a sensitivity of 0.826. The AUC for predicting coronary function with MLA < 2.74 mm2 was 0.732, with an accuracy of 79.3% and a specificity of 0.928. Compared with previous studies, the innovation of this study is the introduction of a new parameter, i.e., the vascular LL. Together, the PB and LL can predict the function of coronary arteries better than any single parameter. The AUC of the combination was 0.862, with a diagnostic accuracy, sensitivity and specificity of 84.8%, 0.826 and 0.725, respectively.

As a single-centre prospective study, this study is limited by the small sample size. The credibility of the results would be improved if further validation could be obtained by a multicentre study with a larger sample size. The study was conducted on individual LAD, LCX, and RCA lesions and did not evaluate LM or tandem lesions. In addition, this study applied IVUS as a method for comparison, which is limited by the resolution and the possibility of subjective error in the measurement of lesions.

## Conclusion

Intermediate coronary lesions, whether they will lead to myocardial ischemia, can be evaluated by IVUS parameters. Single PB, MLA, and LL parameters have a certain predictive value. Combined with PB and LL IVUS parameters can be more accurate than single of them.

### Supplementary Information

**Additional file 1: Table S1.** Comparison of MLA in different states.

**Additional file 2: Table S2.** Comparison of PB in different states.

**Additional file 3: Table S3.** Patient characteristics between the QFR ≤ 0.8 and the QFR > 0.8.

## Data Availability

The datasets used and/or analysed during the current study are available from the corresponding author on reasonable request and with permission of Shanghai general hospital.

## Abbreviations

IVUS: Intravascular ultrasound
QFR: Quantitative fraction ratio
ROC: Receiver operating characteristic curve
PB: Plaque burden
LL: Lesion length
AUC: Area under the curve
PCI: Percutaneous coronary intervention
FFR: Fractional flow reserve
ACE-Is: Angiotensin-converting enzyme inhibitors
ARBs: Angiotensin receptor blockers
MLA: Minimum lumen area
DS: Diameter of stenosis
AS: Area of stenosis
LAD: Left anterior descending
LCX: Artery; left circumflex
RCA: Artery; right coronary artery
PB: Plaque burden
PPVs: Positive predictive values
