# Correction: Predictive value of intravascular ultrasound for the function of intermediate coronary lesions

**DOI:** 10.1186/s12872-023-03545-9

**Published:** 2023-10-27

**Authors:** Yajuan Zhu, Guowei Zhou, Lei Yang, Keng Liu, Yuning Xie, Wenyi Yang, Qiuyan Dai

**Affiliations:** 1 Department of Cardiology, Shanghai General Hospital of Nanjing Medical University, No.100, Haining Rd, Hongkou District, Shanghai, 200080 China; 2 Department of Cardiology, Shanghai General Hospital of Shanghai Jiao Tong University School of Medicine, No.100, Haining Rd, Hongkou District, Shanghai, 200080 China; 3 Department of Emergency, Shanghai General Hospital of Shanghai Jiao Tong University School of Medicine, No.100, Haining Rd, Hongkou District, Shanghai, 200080 China; 4 Menghai County People’s Hospital, Xishuangbanna, Yunnan Province China; 5 School of Oral Medicine, Nanjing Medical University, No.1, Shanghai RD, Nanjing City, Jiangsu Province China

**Correction to: BMC Cardiovascular Disorders (2023) 23:457**

10.1186/s12872-023-03489-0

In this article [1], The Funding information section was missing from this article and should have read “The study received funding from the Science and Technology Commission of Shanghai Municipality (Exploration Project 2021: 21TS1400300). The funding source had no role in 1) the design and conduct of the study; 2) collection, management, analysis, and interpretation of the data; 3) preparation, review, or approval of the manuscript; and 4) decision to submit the manuscript for publication”.

Then the author name “Wen-Yi Yang” was incorrectly written as “Wenyi Yang”

The original article has been corrected.
