# Supplementary material for: Predictive value of intravascular ultrasound for the function of intermediate coronary lesions
Source: BMC Cardiovasc Disord. 2023 Sep 14;23:457. doi: 10.1186/s12872-023-03489-0 (PMC10500773; doi:10.1186/s12872-023-03489-0)
Supplement: Supplementary file 1 — Additional file 1: Table S1. Comparison of MLA in different states. [file 12872_2023_3489_MOESM1_ESM.docx]

**Table S1 Comparison of MLA in different states**

| Variables | MLA | Z or H value | P-Value |
| --- | --- | --- | --- |
| Age, yrs |  | -0.835 | 0.404 |
| <60 (n=17) | 4.10(3.47-5.20) |  |  |
| ≥60 (n=75) | 3.73(3.02-4.92) |  |  |
| Gender |  | -0.377 | 0.706 |
| Male (n=65) | 3.73(2.98-5.01) |  |  |
| Female (n=27) | 3.79(3.29-4.65) |  |  |
| History of HTN |  | -2.113 | 0.035 |
| Yes (n=54) | 3.70(2.97-4.40) |  |  |
| None (n=38) | 4.01(3.48-5.69) |  |  |
| History of DM. |  | -1.852 | 0.064 |
| Yes (n=27) | 3.70(2.77-4.10) |  |  |
| None (n=65) | 3.92(3.13-5.47) |  |  |
| History of smoking |  | -2.276 | 0.023 |
| Yes (n=19) | 3.36(2.54-4) |  |  |
| None (n=73) | 3.81(3.32-5.45) |  |  |
| History of alcohol |  | -0.309 | 0.757 |
| Yes (n=7) | 3.72(2.61-7.43) |  |  |
| None (n=85) | 3.79(3.05-4.92) |  |  |
| BMI |  | -0.618 | 0.537 |
| <24 (n=43) | 3.71(2.77-5.1) |  |  |
| ≥24 (n=47) | 3.81(3.29-4.91) |  |  |
| Vascular |  | 0.532 | 0.766 |
| LAD (n=71) | 3.73(2.94-4.92) |  |  |
| LCX (n=10) | 4.31(3.29-6.70) |  |  |
| RCA (n=11) | 3.79(3.65-4.24) |  |  |

Note: MLA. Minimum lumen area; HTN. Hypertension; DM. [Diabetes](javascript:;) [mellitus](javascript:;); BMI. Body mass index; LAD. Left anterior descending coronary artery; LCX. Left circumflex coronary artery; RCA. Right coronary artery
