# Supplementary material for: Predictive value of intravascular ultrasound for the function of intermediate coronary lesions
Source: BMC Cardiovasc Disord. 2023 Sep 14;23:457. doi: 10.1186/s12872-023-03489-0 (PMC10500773; doi:10.1186/s12872-023-03489-0)
Supplement: Supplementary file 2 — Additional file 2: Table S2. Comparison of PB in different states. [file 12872_2023_3489_MOESM2_ESM.docx]

**Table S2 Comparison of PB in different states**

| Variables | PB | Z or H value | P-Value |
| --- | --- | --- | --- |
| Age, yrs |  | -0.282 | 0.778 |
| <60 (n=17) | 69(50-75.5) |  |  |
| ≥60 (n=75) | 68(61-74) |  |  |
| Gender |  | -0.88 | 0.379 |
| Male (n=65) | 69(60.5-75.5) |  |  |
| Female (n=27) | 68(59-73) |  |  |
| History of HTN |  | -2.079 | 0.038 |
| Yes (n=54) | 70(63.75-76) |  |  |
| None (n=38) | 66(50-73.25) |  |  |
| History of DM |  | -2.236 | 0.025 |
| Yes (n=27) | 74(65-77) |  |  |
| None (n=65) | 68(56-72) |  |  |
| History of smoking |  | -1.656 | 0.098 |
| Yes (n=19) | 72(63-81) |  |  |
| None (n=73) | 68(59-74) |  |  |
| History of alcohol |  | -0.789 | 0.43 |
| Yes (n=7) | 72(56-81) |  |  |
| None (n=85) | 68(60.5-74) |  |  |
| BMI |  | -0.606 | 0.544 |
| <24 (n=43) | 68(56-74) |  |  |
| ≥24 (n=47) | 69(62-77) |  |  |
| Vascular |  | 1.582 | 0.453 |
| LAD (n=71) | 68(61-74) |  |  |
| LCX (n=10) | 62.5(50.75-77.5) |  |  |
| RCA (n=11) | 69(67-76) |  |  |

Note: PB. Plaque burden; HTN. Hypertension; DM. [Diabetes](javascript:;) [mellitus](javascript:;); BMI. Body mass index; LAD. Left anterior descending coronary artery; LCX. Left circumflex coronary artery; RCA. Right coronary artery
