# Supplementary material for: Predictive value of intravascular ultrasound for the function of intermediate coronary lesions
Source: BMC Cardiovasc Disord. 2023 Sep 14;23:457. doi: 10.1186/s12872-023-03489-0 (PMC10500773; doi:10.1186/s12872-023-03489-0)
Supplement: Supplementary file 3 — Additional file 3: Table S3. Patient characteristics between the QFR ≤ 0.8 and the QFR > 0.8. [file 12872_2023_3489_MOESM3_ESM.docx]

| **Table S3. Patient characteristics between the QFR ≤ 0.8 and the QFR > 0.8** | | | | | |
| --- | --- | --- | --- | --- | --- |
|  | **B** | **SE** | **Wald** | **P** | **OR** |
| HTN | 0.623 | 0.514 | 1.471 | 0.225 | 1.865 |
| SEX | 0.856 | 0.505 | 2.871 | 0.09 | 2.353 |
| AGE | -0.026 | 0.025 | 1.153 | 0.283 | 0.974 |
| BMI | 0.067 | 0.07 | 0.913 | 0.339 | 1.069 |
| DM | 0.069 | 0.525 | 0.017 | 0.895 | 1.072 |
| SMOKING | 0.419 | 0.566 | 0.548 | 0.459 | 1.52 |
| DRINKING | 0.198 | 0.874 | 0.051 | 0.821 | 1.219 |
| VESSEL | -0.861 | 0.523 | 2.708 | 0.1 | 0.423 |

Note: B. Regression coefficient; SE. Standard error; Wald. Chi-squared value; OR. Odds ratio;
